# Sustained Performance of Cardiac Arrest Prevention in Pediatric Cardiac Intensive Care Units

**DOI:** 10.1001/jamanetworkopen.2024.32393

**Published:** 2024-09-09

**Authors:** Dana Mueller, David K. Bailly, Mousumi Banerjee, R.A. Bertrandt, Santiago Borasino, Mario Briceno-Medina, Titus Chan, J. Wesley Diddle, Yuliya Domnina, Katherine Clarke-Myers, Chloe Connelly, Amy Florez, Michael Gaies, Janie Garza, Rod Ghassemzadeh, John Lane, Amy N. McCammond, Mary K. Olive, Laura Ortmann, Parthak Prodhan, Tia Tortoriello Raymond, Jun Sasaki, Carly Scahill, Luke W. Schroeder, Kurt R. Schumacher, David K. Werho, Wenying Zhang, Jeffrey Alten

**Affiliations:** 1 Department of Pediatrics, Division of Cardiology, University of California, San Diego, Rady Children’s Hospital, San Diego; 2 Department of Pediatrics, Division of Pediatric Critical Care, University of Utah, Primary Children’s Hospital, Salt Lake City; 3 Department of Biostatistics, University of Michigan, Ann Arbor; 4 Department of Pediatric Critical Care, Medical College of Wisconsin, Children’s Wisconsin, Milwaukee; 5 Department of Pediatrics, University of Alabama at Birmingham, Cardiac Critical Care, Birmingham; 6 Department of Pediatrics, Heart Institute, University of Tennessee, Le Bonheur Children’s Hospital, Memphis; 7 Department of Pediatrics, Division of Cardiac Critical Care, University of Washington, The Heart Center, Seattle Children’s Hospital, Seattle; 8 Department of Anesthesiology and Critical Care Medicine, Children’s Hospital of Philadelphia, Philadelphia, Pennsylvania; 9 Division of Cardiac Critical Care Medicine, Children’s National Hospital, Washington, DC; 10 Department of Pediatrics, University of Cincinnati School of Medicine, Heart Institute, Cincinnati Children’s Hospital, Cincinnati, Ohio; 11 Department of Pediatrics, Cardiac Critical Care, Medical City Children’s Hospital, Dallas, Texas; 12 Department of Critical Care Medicine, Cardiac Intensive Care Unit, UPMC Children’s Hospital of Pittsburgh, University of Pittsburgh, Pittsburgh, Pennsylvania; 13 Division of Cardiovascular Intensive Care, Phoenix Children’s Hospital, Phoenix, Arizona; 14 Department of Pediatrics, Pediatric Cardiac Intensive Care, University of California, San Francisco, Benioff Children’s Hospital, San Francisco; 15 Department of Pediatrics, Division of Pediatric Cardiology, University of Michigan Medical School, C.S. Mott Children’s Hospital, Ann Arbor; 16 Department of Pediatrics, University of Nebraska Medical Center, Children’s Nebraska, Omaha; 17 Department of Pediatrics, Division of Pediatric Cardiology, University of Arkansas for Medical Sciences, Arkansas Children’s Hospital, Little Rock; 18 Department of Cardiology, Boston Children’s Hospital, Harvard Medical School, Boston, Massachusetts; 19 Department of Pediatrics, Heart Institute, Children’s Hospital Colorado, Aurora; 20 Department of Pediatrics, Medical University of South Carolina, Charleston

## Abstract

**Importance:**

The Pediatric Cardiac Critical Care Consortium (PC4) cardiac arrest prevention (CAP) quality improvement (QI) project facilitated a decreased in-hospital cardiac arrest (IHCA) incidence rate across multiple hospitals. The sustainability of this outcome has not been determined.

**Objective:**

To examine the IHCA incidence rate at participating hospitals after the QI project ended and discern which factors best aligned with sustained improvement.

**Design, Setting, and Participants:**

This observational cohort study compared IHCA data from the CAP era (July 1, 2018, to December 31, 2019) with data from the 2-year follow-up era (March 1, 2020, to February 28, 2022). Data were obtained from pediatric cardiac intensive care units (CICUs) from 17 PC4 CAP–participating hospitals.

**Intervention:**

The CAP practice bundle was designed to facilitate local practice integration, with the intention to implement, adapt, and continue CAP processes beyond the CAP era. A web-based survey was administered 2 years after the end of the project to estimate CAP-specific QI work.

**Main Outcomes and Measures:**

Risk-adjusted IHCA incidence rates across all admissions were compared between study eras. The survey generated a novel hospital-specific QI sustainability score, which is generally reflective of the sum of local CAP work performed.

**Results:**

There were no clinically important differences in demographic and admission characteristics between the 13 082 CAP era admissions and 16 284 follow-up admissions (total mean [SD] age, 5.1 [8.4] years; 56.1% male). Risk-adjusted IHCA incidences were not different between the CAP vs follow-up eras (2.8% vs 2.8%; odds ratio, 1.03; 95% CI, 0.89-1.19), suggesting sustained prevention improvement. There was also no difference between eras in risk-adjusted IHCA incidence within medical, surgical, or high-risk subgroups. A lower hospital QI sustainability score was correlated with higher odds for IHCA in the follow-up vs CAP era (correlation coefficient, −0.58; *P* = .02). Five hospitals had increases of 1% or greater in risk-adjusted IHCA rates in the follow-up era; these hospitals had significantly lower QI sustainability scores and were less likely to have adopted sustainability elements during the CAP era or report persistent engagement for CAP-related QI processes during follow-up.

**Conclusions and Relevance:**

In this cohort study of all CICU admissions across 17 hospitals, IHCA prevention was feasible and sustainable; the established reduction in risk-adjusted IHCA rate was maintained for at least 2 years after the end of the CAP project. Both implementation strategies and continued engagement in CAP processes during the follow-up era were associated with sustained improvement.

## Key Points

**Question:**

Can 17 hospitals maintain the reduced in-hospital cardiac arrest (IHCA) rate they achieved during the multicenter cardiac arrest prevention (CAP) quality improvement project, and, if so, what factors are associated with sustained improvement?

**Findings:**

In this cohort study of 13 082 CAP era admissions and 16 284 follow-up admissions, there was no difference in risk-adjusted IHCA incidence between the CAP project era and 2-year follow-up era, suggesting sustained improvement. Hospitals with waning engagement in improvement processes had higher odds of IHCA in the follow-up era.

**Meaning:**

These findings suggest that IHCA prevention improvement is sustainable; consideration of sustainability during the implementation stage and continued engagement in cardiac arrest prevention practices may be associated with maintenance of lower IHCA rate.

## Teaser

This cohort study of pediatric cardiac intensive care unit admissions explores whether an in-hospital cardiac arrest incidence reduction was maintained during a 2-year follow-up period among hospitals that engaged in a cardiac arrest prevention quality improvement project.

## Introduction

The Pediatric Cardiac Critical Care Consortium (PC4) cardiac arrest prevention (CAP) project was a successful multicenter, collaborative quality improvement (QI) initiative that led to a 30% reduction in risk-adjusted in-hospital cardiac arrest (IHCA) incidence rate during the 18-month project compared with the baseline IHCA rate for all cardiac intensive care unit (CICU) admissions at 15 participating hospitals.^1^ This finding is the strongest support yet for the recent paradigm shift in pediatric resuscitation science that suggests some IHCAs in pediatric ICUs are preventable and that IHCA rate is a modifiable target.^2,3^ However, it was unclear whether participating hospitals were capable of sustaining these outcomes once the CAP project and collaborative learning network concluded.

Sustainability capacity is an organization’s ability to implement and maintain change,^4^ which remains an elusive goal when implementing single-center and multicenter QI initiatives because many participants watch initial success regress to baseline once the formal project ends.^5^ Recognized barriers for sustainment include waning levels of staff engagement, insufficient implementation efforts geared toward sustainability, and lack of administrative support for QI practices.^4,6^ We designed the CAP project to be a sustainable intervention, aiming toward persistent reduction in IHCA rates across participating institutions after project end. In this study, we sought to explore whether IHCA incidence reduction was maintained in a follow-up era 2 years after project end among 17 hospitals that fully implemented and engaged in the CAP QI project. We also sought to understand specific processes used during CAP implementation and/or during the follow-up era that were most closely aligned with maintained reduction in the IHCA rate. Finally, we assessed associations between CAP sustainability processes and IHCA rates.

## Methods

### Study Populations and Setting

The PC4 is a QI collaborative composed of more than 75 hospitals aiming to improve outcomes in patients with critical pediatric cardiovascular disease. The PC4 data infrastructure has previously been described.^7,8^ Hospitals enter data on every CICU admission, including IHCA details. The University of Michigan Institutional Review Board approved this project with waiver of informed consent because there was no patient contact and minimal risk of confidentiality loss. This study represents observational cohort analyses of hospitals that participated in the PC4 CAP QI project. The PC4 registry data (demographic and IHCA variables) for all admissions within 17 hospital CICUs were compared between the initial 18-month CAP QI project era from July 1, 2018, to December 31, 2019, and the 2-year CAP follow-up era from March 1, 2020, to February 28, 2022. This study follows Strengthening the Reporting of Observational Studies in Epidemiology (STROBE) reporting guideline.^9^

### CAP Project

Details of the PC4 CAP QI project have been previously published.^1^ In brief, volunteer CICU teams formed a collaborative learning network led by QI specialists and physician and nurse faculty to implement a CAP bundle and engage in IHCA prevention–focused QI work. The CAP bundle had 5 simple elements that promote increased situational awareness and communication among bedside clinicians to recognize and mitigate deterioration in 3 high-risk patient groups (eTable 1 in Supplement 1).

### Sustainability Design

The CAP project was designed to be a sustainable intervention; the project was envisioned to include multiple hospitals gaining skills to independently perform QI work on IHCA prevention and share learning about experiences, successes, and failures. Understanding that CICUs would be different with respect to environment, culture, expertise, and resources to foster change through QI, we sought to have teams learn and understand concepts associated with successful implementation and sustainment of a QI intervention. Sustainability design elements were adapted from the Institute for Healthcare Improvement’s 6 organizational properties associated with sustainability of change.^10^ The CAP processes associated with each of these elements are detailed in Table 1.^11^

**Table 1.** Sustainability Design of the CAP Bundle

Sustainability element	Implementation
Supportive management structure	Recruit local CICU physician and/or nurse to be CAP champion. Engaging local CICU and hospital leadership to support project, particularly through recurrent presentations of local and/or collaborative data demonstrating need for improved IHCA rate. Collaborative CAP presentations made available to all participating centers.
Structures to foolproof change	CAP bundle elements and inclusion criteria were designed to be lightly prescriptive and simple to fit into daily workflow. CICUs encouraged to develop order sets, include patient identification into daily flow huddles and develop signage to incorporate bundle elements more readily.^a^ Successful examples made available to all participating centers.
Robust, transparent feedback systems	CAP teams taught to effectively interpret and display data to track progress. Public data sharing and progress updates encouraged. Real-time data benchmarking of risk-adjusted IHCA rate available on the PC4 platform, which allows individual institutions to easily assess their prevention performance against other PC4 institutions, even after project end.
Shared sense of the systems to be improved	At institutions, multidisciplinary involvement encouraged to take ownership and accountability of the project and results and work toward true system change.^a^ Nurse champions formed separate collaborative to engage bedside nursing staff.^a^
Culture of improvement and deeply engaged staff	Embed CAP project into local culture: build pride, buy-in, empowerment. Tangible efforts: CAP display boards, signage, huddle signs and plans, and nursing bedside tools. Incorporate CAP education into routine staff and onboarding education.^a^
Formal capacity–building programs	CAP teams guided through model for improvement and plan-do-study-act cycles.^a^ QI experts engaged each hospital on demand to help with institution-specific barriers and/or provide further QI education; encouraged local QI expert.^a^ Monthly CAP webinars included shared learning and successes, QI education, and CAP process and IHCA outcome data; goal: to avoid pitfalls and achieve successful implementation.

Abbreviations: CAP, cardiac arrest prevention; CICU, cardiac intensive care unit; IHCA, in-hospital cardiac arrest; PC4, Pediatric Cardiac Critical Care Consortium; QI, quality improvement.

^a^ These items represent CAP era sustainability design elements evaluated by the CAP Site Champion Survey to estimate a site’s commitment to building for sustainability.

### Measuring Engagement With Sustainability Practices

A web-based survey (SurveyMonkey Inc) was administered to CAP physician and/or nurse champions at all 17 hospitals after the follow-up era in February 2023 (eAppendix in Supplement 1). The survey was developed by CAP clinical champions with the intent that it be a surrogate measure of CAP era implementation factors that might be associated with sustainability as well as continued CAP-specific QI engagement during the follow-up era. A QI sustainability score was generated for each hospital and defined as the sum of all questions, with 1 point for all yes responses to binary questions and 1 point for a priori–determined thresholds for Likert-style questions on a 5-point scale (Table 2).^12^ Total possible scores ranged from 0 to 20, with higher scores indicating more engagement in CAP QI processes. When there were multiple survey responses (6 hospitals had 2 respondents and 2 hospitals had 3 respondents), scores for each question were to be averaged, although respondents’ answers led to no discordant scoring for any question.

**Table 2.** CAP Site Champion Survey: Centers With a 1% or Greater Absolute Increase in IHCA Rate in Follow-Up Era vs All Others^a^

Survey question	Follow-up IHCA rate increase <1%, No. (%) (n = 12)	Follow-up IHCA rate increase ≥1%, No. (%) (n = 5)	*P* value
Within the last 1 y, there was/is a formal physician champion of CAP	9 (75)	3 (60)	.54
The CAP physician champion at the beginning of this CAP project still has active role with CAP	7 (58)	1 (20)	.30
Within the last year, there was/is a formal nurse champion for CAP	5 (42)	2 (40)	>.99
At the beginning of the CAP project, was there at least 1 formal nurse or APP co-lead for CAP	8 (67)	2 (40)	.59
The original RN CAP champion still has active role with CAP	5 (42)	0	.24
How would you compare the current CAP processes within the CICU to peak CAP efforts 2 years ago? (ie, is CAP ingrained in the CICU culture and workflow?): at least “maintained the same”	8 (67)	0	.03
Has there been any of the following: formal unit-wide education or updates or introduction of new CAP processes in last 2 y?	8 (67)	2 (40)	.59
Is there a CAP order set or other CAP processes that are ordered and viewed within the EMR? (must have been created after CAP started)	7 (58)	2 (40)	>.99
Does your center have CAP processes in place that were not part of original CAP protocol, but DID NOT exist before CAP? (ie, center-specific CAP criteria [ie, pulmonary hypertension, heart failure, high vasoactive inotropic score, etc]; different emergency medications at bedside; different bundle elements; etc)	9 (75)	1 (20)	.10
There is a heart institute– or hospital-supplied quality or safety specialist that has an active defined role with CAP at your center in the last 2 y	7 (58)	1 (20)	.29
Training or education on CAP processes are incorporated into our new hire onboarding processes	7 (58)	0	.04
Training or education on CAP processes are incorporated into our ongoing unit-based education	8 (67)	2 (40)	.59
A multidisciplinary CAP team is still active in our CICU	9 (75)	1 (20)	.10
How often does your CAP team still engage in reviewing compliance for eligible patient inclusion in CAP: at least “regularly, but not consistently”	8 (67)	1 (20)	.13
How often does your CAP team still engage in reviewing compliance for the various bundle elements for included patients: at least “regularly, but not consistently”	5 (42)	0	.24
How often does your CAP team still engage in reviewing CAP processes and adjusting as needed (PDSA cycles): at least “regularly, but not consistently”	5 (42)	0	.24
How often does your CAP team still engage in reviewing IHCA metrics as a team: at least “regularly, but not consistently”	8 (67)	1 (20)	.13
How often does your CAP team still engage in reviewing events/debriefing: at least “regularly, but not consistently”	11 (92)	5 (100)	>.99
How often does your CAP team still engage in reporting back to staff after a debrief or PDSA cycle: at least “regularly, but not consistently”	7 (58)	2 (40)	.62
How often does your CAP team still engage in observing CAP rounds and offering feedback: at least “regularly, but not consistently”	0	0	>.99

Abbreviations: APP, advanced practice provider; CAP, cardiac arrest prevention; CICU, cardiac intensive care unit; EMR, electronic medical record; IHCA, in-hospital cardiac arrest; PDSA, plan-do-study-act; RN, registered nurse.

^a^ One point was given for each yes response for all questions, adapted from the survey; the sum of all yes responses represented each center’s quality improvement sustainability score. See complete survey in the eAppendix in Supplement 2.

### Statistical Analysis

In-hospital cardiac arrest was defined according to the American Heart Association’s Get With the Guidelines–Resuscitation as any episode that required chest compressions and/or defibrillation and elicited resuscitation response and documentation.^13^ High risk was defined as meeting any 1 of 3 CAP inclusion criteria (eTable 1 in Supplement 1).

Admission and patient characteristics between the CAP and follow-up eras were analyzed for differences using χ^2^ testing (eTable 2 in Supplement 1). Risk-adjusted IHCA rates for each era were calculated by fitting multivariable logistic regression models based on patient-level IHCA variables identified in our previous work (variables in models include age, chromosomal anomalies or syndrome, underweight status, preoperative risk factors, surgical complexity score, acute heart failure diagnosis, mechanical ventilation in the first hour, and lactate level >3 mg/dL [to convert to millimoles per liter, multiply by 0.111]).^14^ Odds ratios (ORs) were calculated comparing rates in the CAP vs follow-up eras. Comparisons were analyzed for all admissions and surgical and medical subgroups (Table 2); there was no risk adjustment for high-risk patients. We applied a 2-level multivariable logistic regression with a hospital-level random effect to account for the heterogeneity across hospitals. The entire cohort was also stratified by hospital, risk-adjusted IHCA was plotted for both eras, and hospital-level IHCA change was assessed using the paired *t* test (eFigure 2 in Supplement 1). We complemented traditional analysis by creating a statistical process control *P* chart for monthly risk-adjusted IHCA incidence during the 2 eras. A 2-sided *P* < .05 was considered statistically significant.

Given the admission sample size at each hospital, there could be a numerical increase in IHCA rate without demonstrating a statistically significant change; therefore, an increase in risk-adjusted IHCA rate of 1% or greater above CAP era baseline was a priori chosen as potentially clinically meaningful when assessing sustained improvement within individual hospitals. To identify organizational and hospital-level QI engagement factors associated with sustained improvement, we used χ^2^ testing to compare each survey question between the cohort of hospitals with an increase in IHCA during the follow-up era of 1% or greater (n = 5) with the other 12 hospitals that more clearly maintained CAP performance. The overall QI sustainability score for each hospital was calculated as described previously and compared using χ^2^ tests. Finally, hospitals were stratified by the median QI sustainability score, and risk-adjusted IHCA rates were compared between strata.

## Results

### Study Era CICU Admissions

Both CAP era data and follow-up era data were available for 29 366 patients from 17 PC4 CAP–participating hospitals. The patient demographic and admission characteristics are shown in eTable 2 in Supplement 1. Females represented 43.9% of the patient population; 56.1% were males. A total of 3.2% of patients were Asian, 14.7% were Black, 62.4% were White, 13.2% were of other race (including American Indian or Alaska Native, Pacific Islander, and multiracial), and 6.5% had unknown race; 18.9% were Hispanic, 77.8% non-Hispanic, and 3.2% of unknown ethnicity. The mean (SD) age was 5.1 (8.4) years. Children younger than 1 year represented slightly more than half of admissions in both eras; 7.1% were adults. Surgical patients represented 57.5% of admissions in both eras. There were no clinically important differences between eras, including proportions of encounters meeting CAP high-risk criteria.

### Sustaining the IHCA Incidence Rate

Table 3 compares IHCA data between eras. There were 13 082 CICU admissions in the CAP era; 352 patients (2.7%) incurred 445 IHCAs. The follow-up era had 16 284 CICU admissions; 459 patients (2.8%) incurred 564 IHCAs. The risk-adjusted IHCA incidence rate for all admissions was similar between eras (2.8% for both; OR, 1.03; 95% CI, 0.89-1.19). There was also no significant change in risk-adjusted IHCA incidence in medical, surgical, or high-risk subgroups. eFigure 1 in Supplement 1 shows a run chart with no temporal change in risk-adjusted IHCA incidence rate after conclusion of the CAP project.

**Table 3.** IHCA Metrics by Admission Type in the CAP vs Follow-Up Era

Metric	CAP era	Follow-up era	OR (95% CI)
**All patients (N = 29 366)**			
No. of admissions	13 082	16 284	NA
Total No. of IHCA episodes	445	564	NA
No. of IHCA episodes per month	25	23	NA
No. (%) of patients with ≥1 IHCA episode	352 (2.7)	459 (2.8)	NA
Risk-adjusted IHCA rate, %	2.80	2.80	1.03 (0.89-1.19)
**High-risk patients (n = 4769)**			
No. of patients	2104	2665	NA
Total No. of IHCA episodes	232	250	NA
No. of IHCA episodes per month	13	10	NA
No. (%) of patients with ≥1 IHCA episode	172 (8.2)	205 (7.7)	0.93 (0.75-1.15)
**Surgical patients (n = 16 895)**			
No. of patients	7495	9400	NA
Total No. of IHCA episodes	296	379	NA
No. of IHCA episodes per month	16	16	NA
No. (%) of patients with ≥1 IHCA episode	235 (3.1)	308 (3.3)	NA
Risk-adjusted IHCA incidence rate, %	3.2	3.3	1.02 (0.86-1.23)
**Medical patients (n = 12 471)**			
No. of patients	5587	6884	NA
Total No. of IHCA episodes	149	185	NA
No. of IHCA episodes per month	8	8	NA
No. (%) of patients with ≥1 IHCA episode	117 (2.1)	151 (2.2)	NA
Risk-adjusted IHCA incidence rate, %	2.1	2.2	1.04 (0.81-1.34)

Abbreviations: CAP, cardiac arrest prevention; IHCA, in-hospital cardiac arrest; NA, not applicable; OR, odds ratio.

### Hospital-Level IHCA Data

eFigure 2 in Supplement 1 shows changes in the risk-adjusted IHCA incidence rate between eras at each hospital. Sustained improvement was demonstrated, with statistically similar IHCA rates between eras in all but 2 hospitals. Nine CICUs had either the same or lower IHCA rates in the follow-up era, although improvement was not statistically significant. Eight CICUs had a higher IHCA rate in the follow-up era, 5 of which experienced an increase 1% or greater, including 2 with statistical significance (Table 4).

**Table 4.** Comparison of QI Sustainability Score and Risk-Adjusted IHCA Incidence Rate Change in the CAP vs Follow-Up Era

Hospital No.	CAP IHCA rate, %	Follow-up IHCA rate, %	Risk-adjusted OR (95% CI) for IHCA in follow-up vs CAP era	*P* value	QI sustainability score
1	3.7	2.0	0.54 (0.29-1.01)	.05	9
2	2.5	1.6	0.59 (0.25-1.43)	.25	18
3	1.6	1.0	0.63 (0.27-1.46)	.29	7
4	3.2	2.2	0.66 (0.41-1.06)	.08	17
5	3.6	2.6	0.70 (0.37-1.33)	.28	13
6	3.0	2.2	0.71 (0.40-1.26)	.25	18
7	3.2	2.4	0.74 (0.45-1.21)	.23	12
8	3.4	3.2	0.91 (0.39-2.11)	.82	12
9	3.3	3.3	0.98 (0.58-1.66)	.94	17
10	2.4	2.8	1.16 (0.59-2.25)	.67	3
11	2.8	3.3	1.18 (0.74-1.89)	.50	6
12	2.4	2.9	1.22 (0.67-2.23)	.51	8
13	3.9	4.9	1.29 (0.72-2.32)	.39	2
14	2.7	4.2	1.58 (0.93-2.67)	.09	6
15	1.6	2.9	1.67 (0.71-3.94)	.24	8
16	1.4	3.0	2.48 (1.16-5.32)	.02	5
17	2.1	5.2	2.75 (1.32-5.74)	.01	5

Abbreviations: CAP, cardiac arrest prevention; IHCA, in-hospital cardiac arrest; OR, odds ratio; QI, quality improvement.

### Hospitals With Highest Increase in Follow-Up IHCA Rate vs All Others

Twenty-seven surveys were completed; all hospitals were represented. Surveys revealed the 5 hospitals with a 1% or greater increase in follow-up IHCA rate had lower frequencies of every metric representing implementation of sustainability strategies during the CAP era compared with the other 12 hospitals, including creation of electronic medical record order sets, adaptation of CAP bundle or CAP inclusion criteria, QI safety specialist on the team, and incorporation of CAP into unit education (Table 2). These 5 hospitals also had lower frequencies of every reported metric consistent with engagement in CAP-related sustainability processes during the follow-up era (Table 2). The CAP processes in follow-up era were not, at minimum, maintained the same in any of these 5 hospitals compared with 8 of the 12 other hospitals that qualitatively maintained the same QI work effort in the follow-up era vs CAP era (*P* = .03).

### Cumulative QI Sustainability Score and Follow-Up IHCA Rate

Each hospital’s cumulative QI sustainability score is given in Table 4. The 5 hospitals that had a 1% or greater increase in follow-up risk-adjusted IHCA had significantly lower QI sustainability scores (eFigure 3 in Supplement 1). For all hospitals, a lower QI sustainability score was associated with higher odds of IHCA in the follow-up vs CAP era (correlation coefficient, −0.58; *P* = .02) (eFigure 4 in Supplement 1).

The median QI sustainability score was 8 (IQR 6-13); the cohort of hospitals above the median threshold (n = 8) had a 24% decrease in their risk-adjusted IHCA incidence rate in the follow-up era compared with the CAP era (2.4% vs 3.2%; OR, 0.74; 95% CI, 0.60-0.91), whereas the cohort of hospitals with QI sustainability scores of 8 or lower (n = 9) had a 38% increase in IHCA rate during the follow-up era (3.2% vs 2.3%; OR, 1.42; 95% CI, 1.16-1.75) (Figure). The 2 CICUs with statistically higher odds for IHCA during the follow-up period both had a QI sustainability score of 5.

**Figure.**
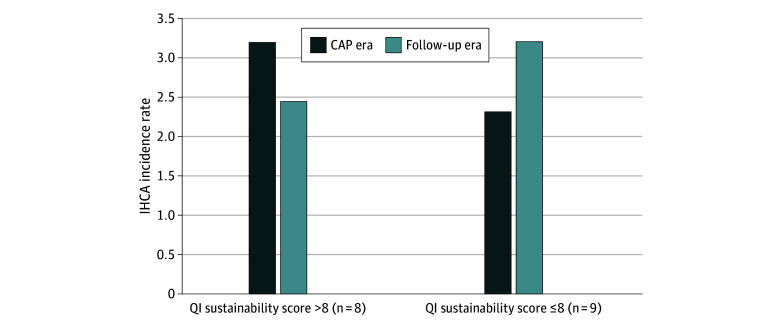
In-Hospital Cardiac Arrest (IHCA) Incidence Rate During the Cardiac Arrest Prevention (CAP) and Follow-Up Eras vs Quality Improvement (QI) Sustainability Score Higher QI sustainability scores in the follow-up era were associated with a statistically lower IHCA incidence rate and vice versa.

## Discussion

Our analysis demonstrates sustainability of CAP practices in CICUs for at least 2 years after the CAP project ended. Importantly, hospital-level data suggest an association between CAP-specific processes (during and after implementation) and maintenance of IHCA prevention performance. These data represent further evidence to support the effectiveness of the PC4 CAP bundle and that continued engagement in CAP-specific QI processes is necessary for sustained improvement. Processes put in place to foster sustainability at the beginning of this project succeeded in most but not all hospitals, and it is important to understand these modifiable factors.

### Project Leadership, QI Engagement, and Sustainability

Sustainability of a health care QI initiative portends that improved outcomes will persist in the face of barriers, including staff or organizational turnover and waning team enthusiasm.^15^ Although the aggregate data demonstrated sustained IHCA reduction, it is worthwhile to recognize variation at the hospital level and to examine the hospitals that had an increased IHCA rate of at least 1% in the follow-up era, suggesting a lack of sustained improvement. None of these hospitals described their CAP-related efforts in the follow-up era as at least maintained compared with the CAP era, and only 1 still had an active multidisciplinary team beyond just a physician leader. Furthermore, the original CAP physician or nurse champion was less likely to be in that role during the follow-up era at these 5 hospitals compared with the other 12 hospitals with more clearly sustained lower IHCA rates, which had much higher frequencies of original CAP champions still active. These data suggest some modes of sustainability failure include transition of project leadership, loss of momentum, or faltering of a shared improvement mental model, all of which can deter team enthusiasm and engagement and be detrimental to project sustainability.^15,16^

### Planning for Sustainability During CAP Implementation

Threats to sustainability are not unique to CAP, so we sought to mitigate threats during the implementation phase in several ways, adapted from the Institute for Healthcare Improvement’s Getting Started Kit: Sustainability and Spread.^10^ Recorded monthly CAP webinars and resources (eg, visual aids and huddle sheets) were easily accessible on the PC+ web-based platform. This virtual CAP toolkit change package was designed to allow any new PC4 hospitals to implement the CAP intervention in their CICUs and enabled any new CAP champions at participating hospitals to catch up. Although this strategy likely contributed to some hospitals’ sustainability despite leader turnover, a more intentional and standardized process addressing concepts of effective QI leadership, including transfer of leadership, may have promoted improved sustainability.^17^

Each of the monthly webinars included targeted QI science education. If feasible, hospitals were also encouraged to recruit a local QI science expert to be a member of their CAP team to reinforce this education and increase sustainability success. In addition to these webinars, all hospitals were provided on-demand access to QI specialists to help the CAP framework fit unique local workflows. The CICUs that maintained their improved IHCA rate showed more mastery of plan-do-study-act (PDSA) work during the CAP era by implementing processes that extended beyond the mandatory CAP framework, such as introducing new high-risk criteria or new bundle elements, work that continued into the follow-up era. In contrast, only 1 of the hospitals with a 1% or higher follow-up IHCA rate implemented more than the minimal requirements for participation. These data support the importance of building an improvement project on a foundation of QI knowledge during implementation (and/or recruiting QI experts to the team) for sustained improvement.^5^

Another important threat to sustainability is the extra time and resources required to perform QI processes. Finding strategies to minimize the perceived burden of extra work, such as integration into normal workflow, may promote project success and likelihood of sustainability. According to our participants, performing the CAP bundle, specifically the twice-daily situational awareness huddles, can be perceived as burdensome. During the CAP era, 25% of all CICU admissions received the CAP bundle for a mean of 4.4 days, which translated into approximately 59 CAP bundle-days to prevent 1 IHCA.^18^ The amount of time required to complete these processes may be a barrier to easily sustaining efforts, exacerbated by team member turnover and/or staffing shortages. Currently, we are working on improving the specificity of IHCA predictive models to more efficiently target both the highest-risk patients and highest-risk period(s) for IHCA. Bundle elements that are less time-consuming could also be explored in new iterations of the CAP bundle as an effort to improve team efficiency.

The up-front sustainability plan focused on incorporation of CAP processes into the normal CICU workflow. Examples included implementation of CAP order sets, discussion of CAP-eligible patients in daily patient flow huddles, bedside visual cues and safety huddle sheets identifying CAP patients, inclusion of CAP into both new and recurring staff education, and IHCA data transparency for all staff (ie, on CICU dashboards). Adoption of many of these named processes was more likely to have occurred in hospitals that maintained their improved IHCA rates in the follow-up era. Both IHCA prevention and project sustainability are feasible when improvement processes are engrained into local culture, and in the future, we will be more direct about the importance of incorporating these items into local practice and sharing successful examples when onboarding new sites.

### Staff Buy-In and Other Contextual Factors Impacting Sustainability

Given the significance of IHCA reduction as the targeted primary outcome metric, we believed it would be easier to get buy-in from CICU and hospital leadership compared with many other QI initiatives; we offered specific strategies to engage leadership about CAP efforts in early webinars. Across hospitals, there was variability in the degree of leadership support, available resources, and level of QI expertise. The concept of IHCA prevention is a paradigm shift in process, and although belief in the utility of CAP increased, the challenge of staff buy-in was still ubiquitous. Organizational leaders dual functioning as CAP leaders likely had a positive influence on initial and continued CAP project buy-in. Physician and nursing buy-in and engagement, leadership support, and local motivation, expertise, and organizational culture for QI change all represent contextual factors that are important considerations to foster a successful QI intervention.^19^ A well-designed QI project, even with very enthusiastic champions, may be rendered ineffective if implementation is attempted in the wrong context. We surmise that contextual factors represent a primary reason the CAP project was not sustainable in some hospitals. In the future, during the onboarding process, evaluation of each hospital’s potential for sustainability could be measured with validated models, including a granular assessment of contextual factors that may influence success and/or failure of implementation and sustainability.^20^ Targeted interventions to mitigate obstacles to change might increase the likelihood of sustainability.

### Limitations

This study has some limitations. The primary limitation is inferred causation. We cannot definitively rule out unmeasured clinical programmatic changes that may have influenced IHCA rates during the follow-up era. Additionally, other improvement initiatives may have had a direct and/or indirect influence on IHCA. Our novel QI sustainability score is not a validated tool to measure QI effort but rather a general overview of CAP activity; whether it accurately reflects high frequency and adherence to CAP bundle activation in follow-up can only be presumed. As discussed above, the context of the clinical and QI environments (ie, leadership support, QI resources and personnel, QI knowledge, and clinician buy-in for importance and need to change) can limit applicability for improvement and sustainability of CAP at some hospitals. Overcoming these contextual obstacles is an important consideration to improve the future success of this bundle and other QI initiatives.

## Conclusions

This study’s findings suggest that improvement in IHCA prevention is sustainable; building for sustainability during the implementation stage and continued engagement in CAP processes after formal project completion may be associated with maintenance of lower IHCA rates. Identifying and addressing contextual and cultural factors may improve the likelihood of sustainability. The next steps include refining the precision of the CAP bundle inclusion criteria and identifying specific processes and infrastructure associated with sustainability of improvement to disseminate across the consortium as well as other critically ill populations.

## Notes

Supplement 1. **eTable 1.** Overview of Cardiac Arrest Prevention (CAP) Bundle and Inclusion (High-Risk) Criteria **eTable 2.** Demographics and Admission Characteristics **eFigure 1**. Monthly In-Hospital Cardiac Arrest (IHCA) Rate **eFigure 2**. Risk Adjusted In-Hospital Cardiac Arrest (IHCA) Incidence Rate During Cardiac Arrest Prevention (CAP Era) vs Follow-Up Era **eFigure 3.** QI Sustainability Score Stratified by Follow-Up IHCA Rate Increase **eFigure 4**. Correlation of Quality Improvement (QI) Sustainability Score With Odds for In-Hospital Cardiac Arrest (IHCA), Follow-Up vs Cardiac Arrest Prevention (CAP) Era **eAppendix.** Cardiac Arrest Prevention (CAP) Champion Survey

Supplement 2.
Nonauthor Collaborators

Supplement 3.
Data Sharing Statement
