# Supplementary material for: Sustained Performance of Cardiac Arrest Prevention in Pediatric Cardiac Intensive Care Units
Source: JAMA Netw Open. 2024 Sep 9;7(9):e2432393. doi: 10.1001/jamanetworkopen.2024.32393 (PMC11385048; doi:10.1001/jamanetworkopen.2024.32393)
Supplement: Supplement 1. — eTable 1. Overview of Cardiac Arrest Prevention (CAP) Bundle and Inclusion (High-Risk) Criteria eTable 2. Demographics and Admission Characteristics eFigure 1. Monthly In-Hospital Cardiac Arrest (IHCA) Rate eFigure 2. Risk Adjusted In-Hospital Cardiac Arrest (IHCA) Incidence Rate During Cardiac Arrest Prevention (CAP Era) vs Follow-Up Era eFigure 3. QI Sustainability Score Stratified by Follow-Up IHCA Rate Increase eFigure 4. Correlation of Quality Improvement (QI) Sustainability Score With Odds for In-Hospital Cardiac Arrest (IHCA), Follow-Up vs Cardiac Arrest Prevention (CAP) Era eAppendix. Cardiac Arrest Prevention (CAP) Champion Survey [file jamanetwopen-e2432393-s001.pdf]

Supplementary Online Content

Mueller D, Bailly DK, Banerjee M, et al; PC4 CAP Collaborators. Sustained performance of cardiac arrest prevention in pediatric cardiac intensive care units. *JAMA Netw Open*. 2024;7(9):e2432393. doi:10.1001/jamanetworkopen.2024.32393

eTable 1. Overview of Cardiac Arrest Prevention (CAP) Bundle and Inclusion (High-Risk) Criteria

eTable 2. Demographics and Admission Characteristics

eFigure 1. Monthly In-Hospital Cardiac Arrest (IHCA) Rate

eFigure 2. Risk adjusted In-Hospital Cardiac Arrest (IHCA) Incidence Rate During Cardiac Arrest Prevention (CAP Era) vs Follow-Up Era

eFigure 3. QI Sustainability Score Stratified by Follow-Up IHCA Rate Increase

eFigure 4. Correlation of Quality Improvement (QI) Sustainability Score With Odds for In-Hospital Cardiac Arrest (IHCA), Follow-Up vs Cardiac Arrest Prevention (CAP) Era

eAppendix. Cardiac Arrest Prevention (CAP) Champion Survey

This supplementary material has been provided by the authors to give readers additional information about their work.

© 2024 Mueller D et al. *JAMA Network Open.*

eTable 1: Overview of Cardiac Arrest Prevention (CAP) bundle and inclusion (high-risk) criteria

**CAP Bundle**	
Included High Risk Populations	
Neonates s/p Cardiopulmonary Bypass (CPB)	
Infants <4 months s/p non-CPB Single Ventricle Palliation	
Medical Patient Intubated within 4 hours of Admission	
Bundle Element	***Explanation***
CAP Safety Huddle	Formal twice daily huddle to:<br>• Develop a shared mental model for recognition or prevention of early deterioration.<br>• Agreement on mitigation plan(s) for reversal and/or rescue.
Vital sign discussion	Patient specific vital signs discussed to:<br>• Identify clear targets amongst the patient care team.<br>• Recognize signs of early deterioration if deviation occurring.
Discussion of Pre-sedation	Discussion of pre-sedation prior to noxious stimuli to:<br>• Prevent hemodynamic instability related to agitation or pain.
Emergency Medication	Patient-specific epinephrine dose drawn up and available at bedside to:<br>• Readily respond to episodes of acute hypotension.<br>• Rescue patient prior to IHCA.
Formal Code Review	Review any IHCA within 2 weeks (ideally 48 hours) of occurrence to:<br>• Identify any modifiable risk factors and/or etiologies.<br>• Develop improvement efforts to target these identified factors.<br>• Readily disseminate information amongst care team/staff.

© 2024 Mueller D et al. *JAMA Network Open.*

eTable 2: Demographics and admission characteristics

Characteristic	Total N=29,366	CAP Period N=13,082	Post-CAP Period N=16,284	p-value
Age				<0.001†
Pre-term infant (0-30days), n (%)	977 (3.3)	414 (3.2)	563 (3.5)	
Term neonate (0-30days), n (%)	4,799 (16.3)	2,077 (15.9)	2,722 (16.7)	
Infant (31-365days), n (%)	9,683 (33.0)	4,176 (31.9)	5,507 (33.8)	
Child (1-18years , n (%)	11,833 (40.3)	5,494 (42.0)	6,339 (38.9)	
Adult (>18years) , n (%)	2,074 (7.1)	921 (7.0)	1,153 (7.1)	
Female	12,895 (43.9)	5,759 (44.0	7,136 (43.8)	0.73
Race				<0.001†
Asian, n (%)	952 (3.2)	411 (3.1)	541 (3.3)	
Black, n (%)	4,308 (14.7)	1,936 (14.8)	2,372 (14.6)	
White, n (%)	18,328 (62.4)	8,374 (64.0)	9,954 (61.1)	
Other, n (%)	3,866 (13.2)	1,702 (13.0)	2,164 (13.3)	
Unknown , n (%)	1,912 (6.5)	659 (5.0)	1,253 (7.7)	
Ethnicity				<0.001†
Hispanic, n (%)	5,564 (18.9)	2,410 (18.4)	3,154 (19.4)	
Non-Hispanic, n (%)	22,856 (77.8)	10,411 (79.6)	12,445 (76.4)	
Unknown, n (%)	946 (3.2)	261 (2.0)	685 (4.2)	
Surgical Admission STAT Category				0.19†
STAT Category 1-3, n (%)	11,894 (40.5)	5,354 (40.9)	6,540 (40.2)	
STAT Category 4-5, n (%)	5,001 (17.0)	2,141 (16.4)	2,860 (17.6)	
Medical Admissions	12,471 (42.5)	5,587 (42.7)	6,884 (42.3)	0.46
High-Risk CAP Eligible Admissions				0.77†
Neonatal Cardiopulmonary Bypass, n (%)	2,617 (8.9)	1,164 (8.9)	1,453 (8.9)	
Single Ventricle Palliation, n (%)	228 (0.8)	104 (0.8)	124 (0.8)	
Medical Admission requiring Mechanical Ventilation <4h, n (%)	1,924 (6.6)	836 (6.4)	1,088 (6.7)	

•† : multi-category group (more than 2 categories) overall significance test results noted with † Data between CAP and follow-up eras were analyzed for differences using chi-square testing

© 2024 Mueller D et al. *JAMA Network Open.*

eFigure 1: Monthly In-Hospital Cardiac Arrest (IHCA) rate

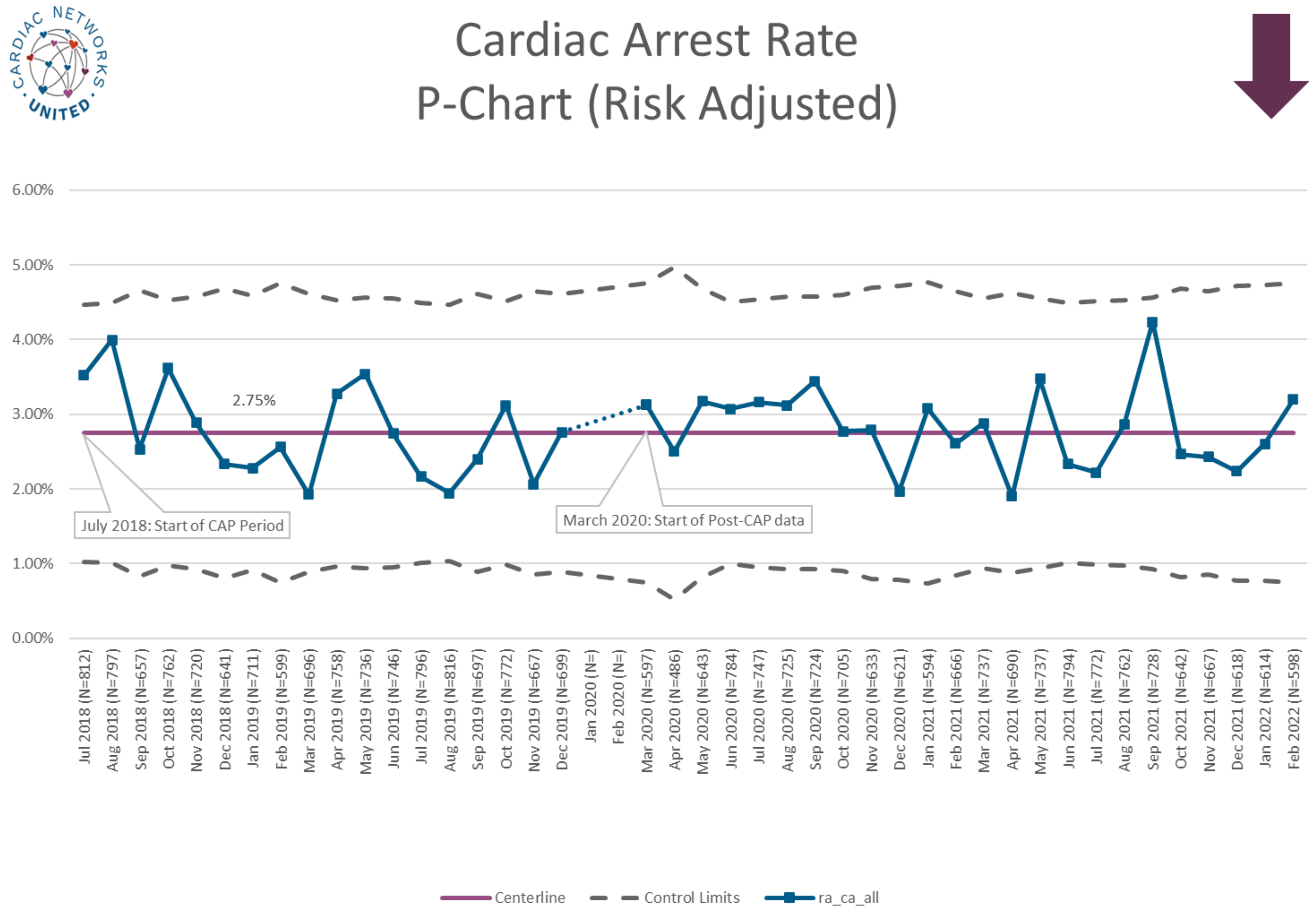

Statistical process control P-chart showing aggregate monthly IHCA rate for all CICU admissions across 17 CAP hospitals across CAP-era and follow-up era. The CAP-era included July, 2018-December, 2019. The follow-up era included data from March, 2020-February, 2022. The median line indicates an aggregate monthly IHCA rate of 2.75% with the dotted lines representing the upper and lower control limits. The arrow indicates the desired direction for change.

© 2024 Mueller D et al. *JAMA Network Open.*

eFigure 2: Risk adjusted In-Hospital Cardiac Arrest (IHCA) incidence rate during Cardiac Arrest Prevention (CAP era) vs follow-up era

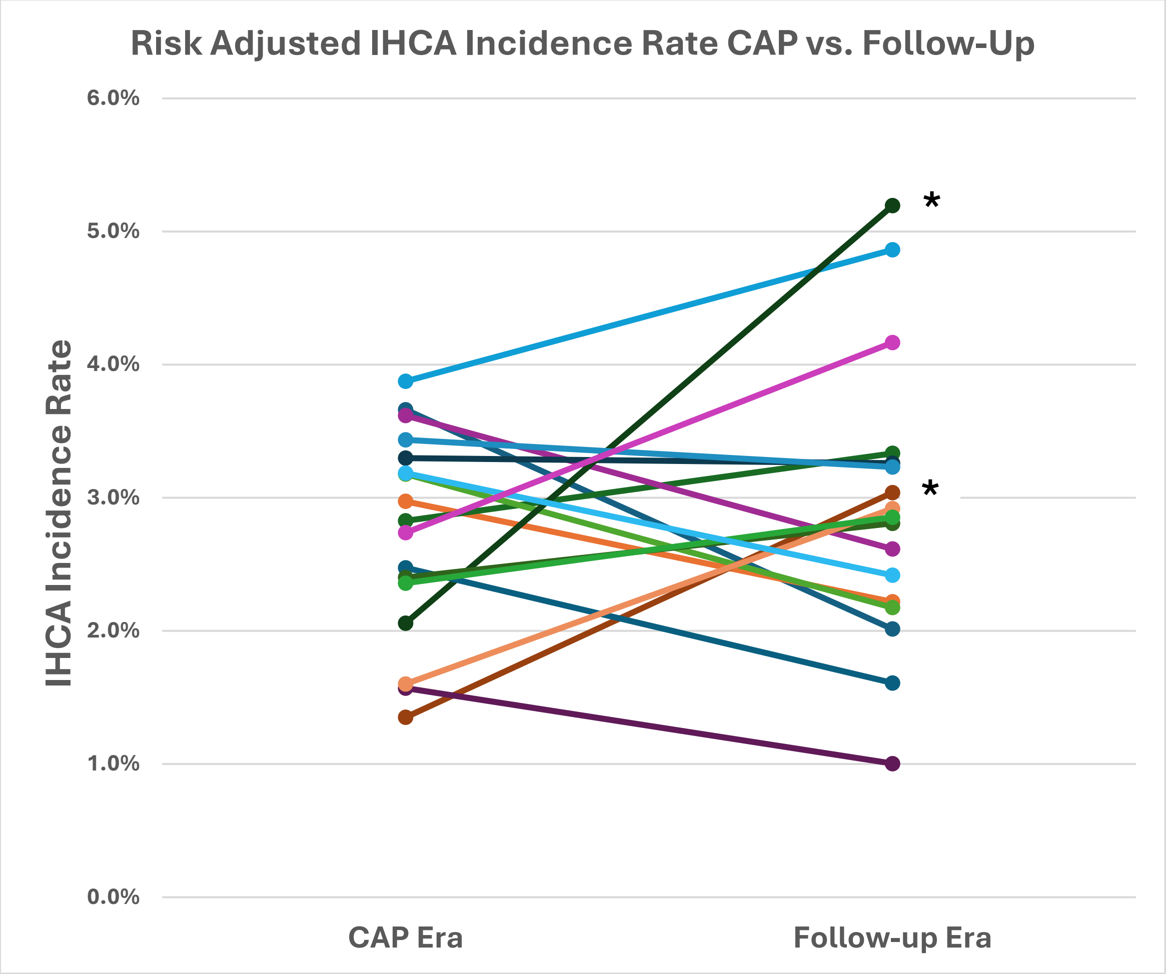

Plot of incidence of in-hospital cardiac arrest between CAP era and follow-up era. Each line represents one of 17 centers; lines designated with * indicate statistically increased odds of IHCA rate in follow-up era.

© 2024 Mueller D et al. *JAMA Network Open.*

eFigure 3. QI Sustainability Score stratified by follow-up IHCA rate increase

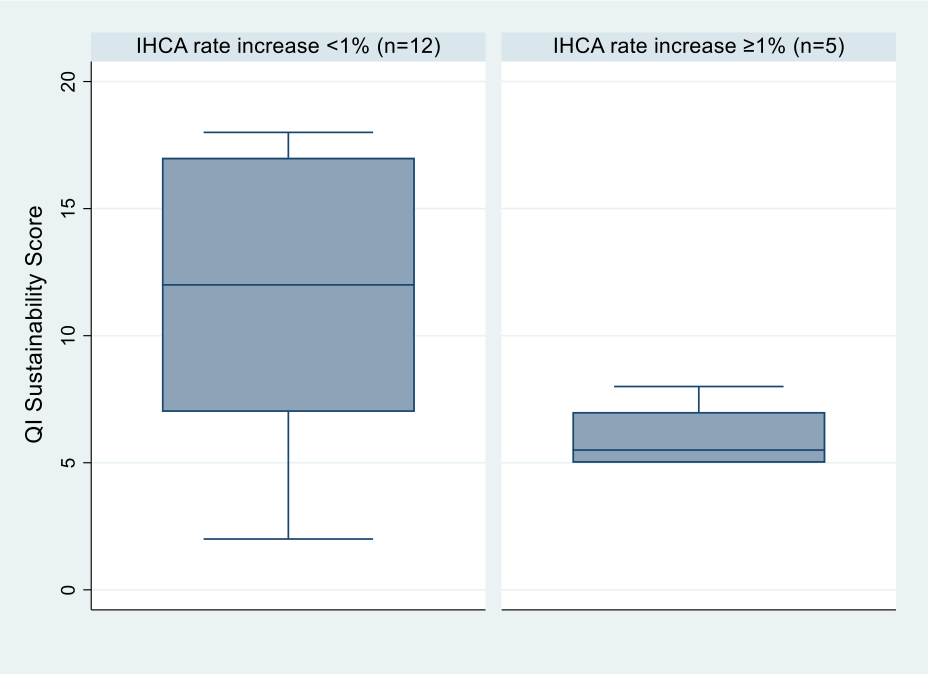

Comparison of Quality Improvement (QI) Sustainability Score in centers with ≥1% absolute increase in risk-adjusted follow-up In-Hospital Cardiac Arrest (IHCA) rate compared to CAP era vs all other centers (<1% increase).

© 2024 Mueller D et al. *JAMA Network Open.*

eFigure 4: Correlation of Quality Improvement (QI) sustainability score with odds for In-Hospital Cardiac Arrest (IHCA), follow-up vs Cardiac Arrest Prevention (CAP) era

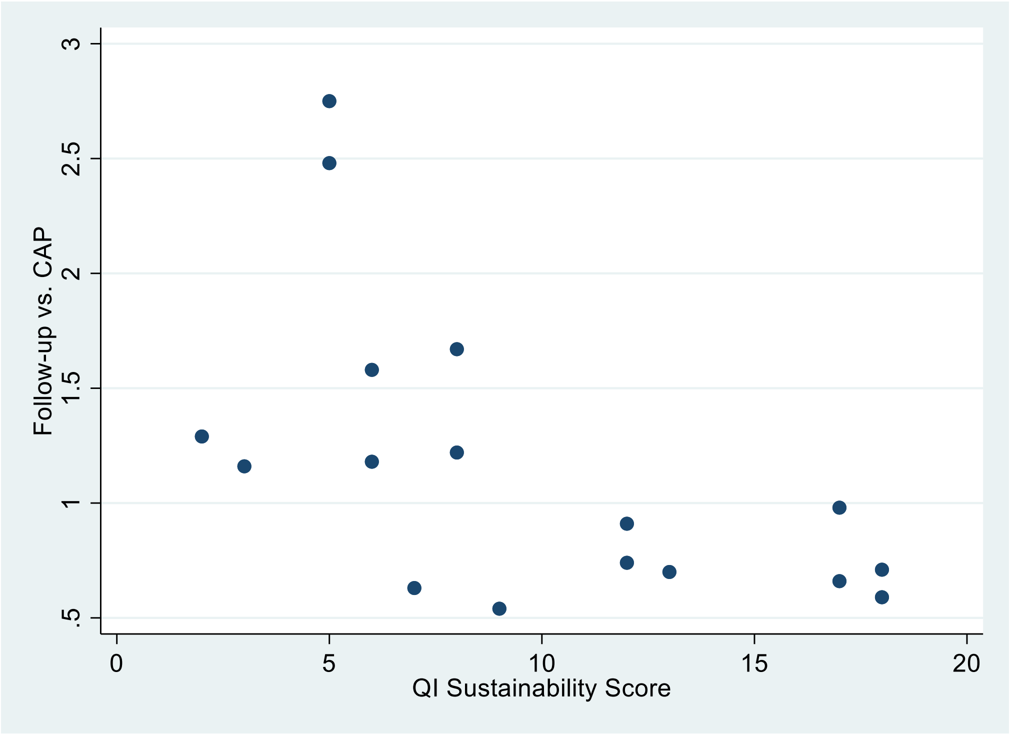

Scatter plot demonstrating correlation of QI sustainability score with odds for IHCA in follow-up era vs. CAP era. Correlation coefficient = -0.58, p=0.018.

© 2024 Mueller D et al. *JAMA Network Open.*

eAppendix. Cardiac Arrest Prevention (CAP) Champion Survey – the web version: **https://www.surveymonkey.com/r/MV5P5VX**

1. You are
    - APP CAP Leader
    - Nurse CAP Leader
    - Physician CAP Leader

2. Your institution

3. Within the last 1 year, there was/is a formal physician champion of CAP
    - Yes
    - No

4. The CAP physician champion **at the beginning of this CAP project**
    - Still has active role with CAP
    - Significantly reduced role with CAP
    - Has left the institution or the CICU

5. Within the last year, there was/is a formal nurse champion for CAP
    - Yes
    - No

6. **At the beginning of the CAP project**, was there at least one formal nurse or APP co-lead for CAP - check all that apply
    - No
    - Nurse leader (charge or manager)
    - Bedside Nurse
    - APP

7. The original RN CAP champion
    - Still has active role with CAP
    - Significantly reduced role with CAP
    - Has left the institution/unit
    - N/A

© 2024 Mueller D et al. *JAMA Network Open.*

8. How would you compare the current CAP processes within the CICU to peak CAP efforts two years ago? (i.e is CAP ingrained in the CICU culture and workflow?)

Continue to adapt and expand
Maintained the same
Starting to fade
Much less
Practically non-existent

9. Has there been any of the following: formal unit-wide Education or Updates or Introduction of new CAP processes in last 2 years?

Yes
No
If Yes, Please Comment

10. Is there a CAP order set or other CAP processes that are ordered and viewed within the EMR? (must have been created after CAP started)

Yes
No
If Yes, Please Comment

11. Does your center have CA prevention processes in place that were not part of original CAP protocol, but **DID NOT exist prior to CAP**? [i.e. center-specific CAP criteria (i.e. pulm htn, heart failure, high VIS, etc.); different emergency medications at bedside; different bundle elements; etc.)

Yes
No
If Yes, Please Comment

12. There is a heart institute or hospital supplied quality or safety specialist that has an active defined role with CAP at your center in the last 2 years.

Yes
No

13. Training or education on CAP processes are incorporated into our new hire on-boarding processes

Yes
No

© 2024 Mueller D et al. *JAMA Network Open.*

14. Training or education on CAP processes are incorporated into our ongoing unit-based education

Yes

No

15. A multidisciplinary CAP team is still active in our CICU

Yes

No

Yes, but only comprises the physician and/or nurse CAP leader

16. For each of the following CAP QI Processes, how often does your CAP team still engage In (Answer Choices: Never; Rarely or Only When Severe/Rare Events Occur; Sometimes or Only After Events or Concerns Raised; Regularly but not Consistently; On a Scheduled/Consistent Basis)

Reviewing compliance for eligible patient inclusion in CAP

Reviewing compliance for the various bundle elements for included patients

Reviewing CAP processes and adjusting as needed (PDSA cycles)

Reviewing metrics as a team

Reviewing events/debriefing

Reporting back to staff after a debrief or PDSA cycle

Observing CAP rounds and offering feedback

Other QI processes related to CAP not mentioned above

Any other comments:

© 2024 Mueller D et al. *JAMA Network Open.*
