# Supplementary material for: Sustained Performance of Cardiac Arrest Prevention in Pediatric Cardiac Intensive Care Units
Source: JAMA Netw Open. 2024 Sep 9;7(9):e2432393. doi: 10.1001/jamanetworkopen.2024.32393 (PMC11385048; doi:10.1001/jamanetworkopen.2024.32393)
Supplement: Supplement 2. — Nonauthor Collaborators [file jamanetwopen-e2432393-s002.pdf]

Supplemental Online Content: Nonauthor Collaborators
*First name, last name, and suffix (if applicable) are required and will appear in PubMed.

*Group Name(s): PC4 CAP Collaborators							
***First Name and Middle Initial(s)**	***Last Name**	***Suffix (eg, Jr, III)**	Academic Degrees	Institution	Location (city, state/province, country)	Role or Contribution, eg, chair, principal investigator	Group (if more than 1 Group listed in the byline) and/or Subgroup (eg, Steering Committee)
Megan A.	Jones		BSN, RN, CCRN	Heart institute, Cincinnati Children's Hospital	Cincinnati, Ohio, USA		
Benjamin	Miller		BSN, RN	Heart institute, Cincinnati Children's Hospital	Cincinnati, Ohio, USA		
Katherine	Wellman		MSN, APRN, CPNP-AC	Heart institute, Cincinnati Children's Hospital	Cincinnati, Ohio, USA		
Alexis	Benscoter		DO	Department of Pediatrics, University of Cincinnati School of Medicine, Heart Institute, Cincinnati Children's Hospital	Cincinnati, Ohio, USA		
David S.	Cooper		MD, MPH	Department of Pediatrics, University of Cincinnati School of Medicine, Heart Institute, Cincinnati Children's Hospital	Cincinnati, Ohio, USA		
Sharyl	Wooton		MS	James M. Anderson Center for Health Systems Excellence, Cincinnati Children's Hospital Medical Center	Cincinnati, Ohio, USA		
Ashley	Moellinger		MSN, CPNP-AC	Cardiovascular Services, Children's of Alabama	Birmingham, Alabama, USA		
Maria	Scalici McAtee		MSN, PNP-AC/PC	Cardiovascular Services, Children's of Alabama	Birmingham, Alabama, USA		
Hayden	Zaccagni		MD	Cardiovascular Services, Children's of Alabama	Birmingham, Alabama, USA		
Janie	Kane		MS APRN PCNS-BC	Arkansas Children's Hospital	Little Rock, Arkansas, USA		
Stephanie	Roper		BSN, RN, CCRN	Arkansas Children's Hospital	Little Rock, Arkansas, USA		
Brittany	Bradley		BSN, RN, CCRN	Arkansas Children's Hospital	Little Rock, Arkansas, USA		
Ashley	Dick		BSN, RN, CCRN	Arkansas Children's Hospital	Little Rock, Arkansas, USA		
Rupal T	Bhakta		MD, MPH	Department of Pediatrics, Division of Pediatric Cardiology, University of Arkansas for Medical Sciences, Arkansas Children's Hospital	Little Rock, Arkansas, USA		
Ashima	Das		MD	Department of Pediatrics, Cardiac Critical Care, Medical City Children's Hospital	Dallas, Texas, USA		
Tejas	Shah		MD	Department of Pediatrics, Cardiac Critical Care, Medical City Children's Hospital	Dallas, Texas, USA		
Grant	Burton		MD	Department of Pediatrics, Cardiac Critical Care, Medical City Children's Hospital	Dallas, Texas, USA		
Mark	Clay		MD	Department of Pediatrics, Cardiac Critical Care, Medical City Children's Hospital	Dallas, Texas, USA		
Gabriela	Centers		MD	Department of Pediatrics, Cardiac Critical Care, Medical City Children's Hospital	Dallas, Texas, USA		
Rudy	Wong		MD, MBA	Department of Pediatrics, Cardiac Critical Care, Medical City Children's Hospital	Dallas, Texas, USA		
Mohammed	Absi		MD	Department of Pediatrics, Heart Institute, University of Tennessee, Le Bonheur Children's Hospital	Memphis Tennessee, USA		
Michelle H.	Grandberry		RN, MSN	Department of Pediatrics, Heart Institute, University of Tennessee, Le Bonheur Children's Hospital	Memphis Tennessee, USA		
Darren	Klugman		MD	Department of Pediatrics, Division of Cardiac Critical Care Medicine, Children's National Hospital,	Washington, DC, USA		
Christine M.	Riley		MSN, CPRN, CPNP-AC	Division of Cardiac Critical Care Medicine, Children's National Hospital	Washington, DC, USA		
Justine M.	Fortkiewicz		MSN, RN-BC, CCRN-K, CPN	Division of Cardiac Critical Care Medicine, Children's National Hospital	Washington, DC, USA		
Lisa A.	Hom		RN, Esq	Children's National Heart Institute, Children's National Hospital	Washington, DC, USA		
Kinjal	Parikh		MD	Division of Cardiac Critical Care Medicine, Nicklaus Children's Hospital	Miami, Florida, USA		
Saleem	Almasarweh		MD	Division of Cardiac Critical Care Medicine, Nicklaus Children's Hospital	Miami, Florida, USA		
Anas	Abdul Kayoum		MD	Division of Cardiac Critical Care Medicine, Nicklaus Children's Hospital	Miami, Florida, USA		
Josh	Koch		MD	Department of Cardiovascular Critical Care, Phoenix Children's Hospital	Phoenix, Arizona, USA		
Amanda	Richardson		RN	Department of Cardiology, Phoenix Children's Hospital	Phoenix, Arizona, USA		

Supplemental Online Content: Nonauthor Collaborators
*First name, last name, and suffix (if applicable) are required and will appear in PubMed.

*First Name and Middle Initial(s)	*Last Name	*Suffix (eg, Jr, III)	Academic Degrees	Institution	Location (city, state/province, country)	Role or Contribution, eg, chair, principal investigator	Group (if more than 1 Group listed in the byline) and/or Subgroup (eg, Steering Committee)
Chasity	Wellnitz		RN, BSN, MPH	Department of Quality Resource Management, Phoenix Children's Hospital	Phoenix, Arizona, USA		
Claudia	Delgado-Corcoran		MD, MPH	Department of Pediatrics, Divisions of Pediatric Critical Care and Palliative Care,	Salt Lake City, Utah, USA		
Trudy	Hardin-Reynolds		DNP, APRN, FNP-BC	Intermountain Healthcare, Primary Children's Hospital	Salt Lake City, Utah, USA		
Thomas B	Do		MD	Department of Pediatrics, Division of Cardiology, University of California San Diego, Rady Children's Hospital	San Diego, California, USA		
Amy J.	Schalke		MSN, CPNP-AC	Department of Nursing, Cardiothoracic Intensive Care Unit, Rady Children's Hospital	San Diego, California, USA		
Siobhán	McMorrow Sciuto		BSN, RN, CCRN	Department of Nursing, Cardiothoracic Intensive Care Unit, Rady Children's	San Diego, California, USA		
Sean	Daley		PharmD, BCPS, BCPPS	Cardiothoracic Intensive Care Unit, Rady Children's Hospital	San Diego, California, USA		
Joshua S.	Wolovits		MD	Department of Pediatrics, University of Texas Southwestern Medical Center, Children's Medical Center Dallas	Dallas, Texas, USA		
Priscilla	Yu		MD	Department of Pediatrics, University of Texas Southwestern Medical Center, Children's Medical Center Dallas	Dallas, Texas, USA		
Julia	Gerstmann		RN	Children's Medical Center Dallas	Dallas, Texas, USA		
Terri	Mannan		BSN, RN, CCRN	Children's Medical Center Dallas and Department of Pediatrics, Cardiac Critical Care, Medical City Children's Hospital	Dallas, Texas, USA		
Jason R.	Buckley		MD	Department of Pediatrics, Division of Pediatric Cardiology, Medical University of South Carolina	Charleston, South Carolina, USA		
Sarah	Tabbutt		MD, PhD	Department of Pediatrics, Pediatric Cardiac Intensive Care, University of California San Francisco, Benioff Children's Hospital	San Francisco, California, USA		
Titus	Chan		MD, MS, MPP	Department of Pediatrics, Division of Critical Care, University of Washington, Seattle Children's Hospital	Seattle, Washington, USA		
Arianna	Davis		MSN, CPNP-AC	Seattle Children's Hospital	Seattle, Washington, USA		
James	Hammel		MD	Children's Hospital and Medical Center	Omaha, Nebraska, USA		
Alyss	Burgert		DNP, RN	Children's Hospital and Medical Center	Omaha, Nebraska, USA		
Bridget	Norton		MD	Department of Pediatrics, University of Nebraska Medical Center, Children's Hospital and Medical Center	Omaha, Nebraska, USA		
Erica	Molitor-Kirsch		MD	Department of Pediatrics, Critical Care Medicine, University of Missouri, Kansas City, Children's Mercy Hospital	Kansas City, Missouri, USA		
Jeremy T	Affolter		MD	Department of Pediatrics, Critical Care Medicine, University of Missouri, Kansas City, Children's Mercy Hospital	Kansas City, Missouri, USA		
Laura	Miller-Smith		MD	Department of Pediatrics, Critical Care Medicine, University of Missouri, Kansas City, Children's Mercy Hospital	Kansas City, Missouri, USA		
Kelly S.	Tieves		DO, MS	Department of Pediatrics, Critical Care Medicine, University of Missouri, Kansas City, Children's Mercy Hospital	Kansas City, Missouri, USA		
Kayla	Walz		ADN, RN	Pediatric Cardiothoracic Intensive Care Unit, C.S. Mott Children's Hospital	Ann Arbor, Michigan, USA		
Colleen	Rosenberg		MSN, RN	Pediatric Cardiothoracic Intensive Care Unit, C.S. Mott Children's Hospital	Ann Arbor, Michigan, USA		
Gabe E.	Owens		MD, PhD	Department of Pediatrics, Division of Pediatric Cardiology, University of Michigan	Ann Arbor, Michigan, USA		
Katherine	Mikesell		BS	PC4 Data Coordinating Center, University of Michigan	Ann Arbor, Michigan, USA		
Eric	Wald		MD, MSCI	of Medicine, Ann & Robert H. Lurie Children's Hospital of Chicago	Chicago, Illinois, USA		
Jade	Clark		BSN, RN, CCRN, CPN, CBC	Department of Pediatrics, Heart Center, Northwestern University Feinberg School of Medicine, Ann & Robert H. Lurie Children's Hospital of Chicago	Chicago, Illinois, USA		
Kimberly	DiMaria		MSN, CPNP-AC	Department of Pediatrics, Heart Institute, Children's Hospital Colorado	Aurora, Colorado, USA		
Ryan	Wilkes		MD	Sanger Heart and Vascular Institute, Levine Children's Hospital	Charlotte, North Carolina, USA		

Supplemental Online Content: Nonauthor Collaborators
*First name, last name, and suffix (if applicable) are required and will appear in PubMed.

*First Name and Middle Initial(s)	*Last Name	*Suffix (eg, Jr, III)	Academic Degrees	Institution	Location (city, state/province, country)	Role or Contribution, eg, chair, principal investigator	Group (if more than 1 Group listed in the byline) and/or Subgroup (eg, Steering Committee)
Page	Steadman		RN, MSN, CPNP-AC	Sanger Heart and Vascular Institute, Levine Children's Hospital	Charlotte, North Carolina, USA		
Amanda L.	Davis		RN, BSN, CCRN	Sanger Heart and Vascular Institute, Levine Children's Hospital	Charlotte, North Carolina, USA		
Amanda	Smith		RN, MSN, CCRN	Sanger Heart and Vascular Institute, Levine Children's Hospital	Charlotte, North Carolina, USA		
Javier J.	Lasa		MD	Department of Critical Care and Cardiology, Baylor College of Medicine, Texas Children's Hospital	Houston, Texas, USA		
Rebecca	Zahn-Schafer		MSN, RN	Department of Critical Care and Cardiology, Baylor College of Medicine, Texas Children's Hospital	Houston, Texas, USA		
Patrick	Maynord		MD	Department of Pediatrics, Division of Pediatric Critical Care Medicine, Vanderbilt University School of Medicine, Monroe Carell Jr. Children's Hospital at Vanderbilt	Nashville, Tennessee, USA		
Andrew	Harold Smith		MD, MSCI, MMHC	Department of Pediatrics, Division of Pediatric Critical Care Medicine, Vanderbilt	Nashville, Tennessee, USA		
Christopher W.	Mastropietro		MD, FCCM	Department of Cardiovascular Surgery and Intensive Care, Riley Children's Hospital	Indianapolis, Indiana, USA		
Monica	Broo		MSN, FNP-C	Department of Cardiovascular Surgery and Intensive Care, Riley Children's Hospital	Indianapolis, Indiana, USA		
Erica	Vinson		FNP-C	Department of Cardiovascular Surgery and Intensive Care, Riley Children's Hospital	Indianapolis, Indiana, USA		
Jane	Kluck		RN-BSN	Children's Wisconsin, Medical College of Wisconsin	Milwaukee, Wisconsin, USA		
Tracy	Baust		BA, CCRC	Department of Critical Care Medicine, UPMC Children's Hospital of Pittsburgh,	Pittsburgh, Pennsylvania, USA		
Tara	Graham		DNP, RN, CNL, CCRN, CPN	UPMC Children's Hospital of Pittsburgh	Pittsburgh, Pennsylvania, USA		
Catherine	Gretchen		MD	UPMC Children's Hospital of Pittsburgh	Pittsburgh, Pennsylvania, USA		
Michael-Alice	Moga		MD, MSc	The Labatt Family Heart Center, University of Toronto, the Hospital for Sick Children	Toronto, Ontario, Canada		
