# Supplementary material for: Sustained Performance of Cardiac Arrest Prevention in Pediatric Cardiac Intensive Care Units
Source: JAMA Netw Open. 2024 Sep 9;7(9):e2432393. doi: 10.1001/jamanetworkopen.2024.32393 (PMC11385048; doi:10.1001/jamanetworkopen.2024.32393)
Supplement: Supplement 3. — Data Sharing Statement [file jamanetwopen-e2432393-s003.pdf]

Data Sharing Statement

Mueller. Sustained Performance of Cardiac Arrest Prevention in Pediatric Cardiac Intensive Care Units. *JAMA Netw Open*. Published September 09, 2024.
doi:10.1001/jamanetworkopen.2024.32393

Data
Data available: No
